# Ectopic orbital meningioma: a retrospective case series

**DOI:** 10.1186/s12886-018-0959-z

**Published:** 2018-11-12

**Authors:** Xiaoming Huang, Dongrun Tang, Tong Wu, Tianming Jian, Fengyuan Sun

**Affiliations:** 1 The School of Medicine, Nankai University, Tianjin, 300071 China; 2 Tianjin Medical University Eye Hospital, Tianjin, 300384 China; 3 Tianjin Orbital Disease Institute, Tianjin, 300384 China

**Keywords:** Ectopic orbital meningioma, Ophthalmic manifestations, Radiographic features, Pathological diagnosis

## Abstract

**Background:**

To evaluate the ophthalmic manifestations and radiographic features of ectopic orbital meningioma to improve diagnostic accuracy.

**Methods:**

Patient data from patients admitted to our institution during a 217-month period from August 1999 to September 2017 were included. Patient ophthalmic manifestations, radiographic features (CT and MRI), diagnosis, pathology, therapeutic regimens, and prognosis were retrospectively reviewed.

**Results:**

Six patients with ectopic orbital meningioma were identified. The mean age at the first visit was 33.2 years (range, 7–56 years). All six patients displayed manifestations of exophthalmos, upper eyelid oedema, and motility impairment with a mean history of illness of 20.3 months (range 3–72 months). Optical lesions were located in the superonasal extraconal compartment (3/6, 50%), bitemporal extraconal compartment (1/6, 16.7%) and orbital intraconal compartment (2/6, 33%). Radiographic features were ill-defined, heterogeneous, enhancing soft tissue masses with extraocular muscular adhesion (6/6, 100%) and calcification (1/6, 16.7%), not adjacent to the optic nerve and not extending along the dura. Six cases were treated intraoperatively with complete surgical resection, indicating that all lesions were independent of the optic nerve and sphenoid ridge. The histopathologic classification was mostly of meningothelial cells (5/6, 83%). Immunohistochemistry revealed EMA and vimentin to have positive expression in all six cases, while two cases were calponin-positive and strongly expressed in the olfactory bulb. Postoperatively, lesions caused no visual impairment, and there were no cases of recurrence.

**Conclusions:**

Ectopic orbital meningiomas are rare tumours that are not easily diagnosed without postoperative histopathology. This report highlights some of the distinguishing features of isolated orbital lesions, especially around the location of frontoethmoidal suture. Accompanying upper eyelid oedema and eye mobility restriction were observed to be dissimilar to other orbital tumours. In these cases, a diagnosis of ectopic orbital meningioma should be considered.

**Electronic supplementary material:**

The online version of this article (10.1186/s12886-018-0959-z) contains supplementary material, which is available to authorized users.

## Background

The meninges have three membranes, including the dura mater, the arachnoid mater, and the pia mater, that envelop the brain and spinal cord. Meningiomas are a variety of tumours caused by arachnoid “cap” cells of meningeal arachnoid villi [1]. Orbital meningiomas can be considered to be primary and secondary in origin [2]. Primary orbital meningioma accounted for 5–10% of all orbital tumours and 30% of all orbital meningiomas; they were also mainly observed in adults and rarely in children [3]. Primary orbital meningiomas originate from the arachnoid layer of the optic nerve sheath. Approximately 70% of orbital meningiomas are secondary intracranial meningiomas, usually originating at the sphenoid ridge, with orbital, intracranial, and intraluminal intrusions [4].

A rare subset of orbital meningiomas that do not involve the optic nerve sheath or sphenoid ridge were initially considered to be “ectopic”. Ectopic orbital meningiomas are occasionally reported as single or multiple case series in the literature. However, there exists a paucity of published clinical evidence regarding the distinguishing features of ectopic orbital meningioma. Preoperative diagnosis is often difficult, which is not conducive to the establishment of surgical methods, surgical operation, and follow-up treatment success.

All cases reported in this report were admitted to the Tianjin Medical University Eye Hospital during a 217-month period. Clinical manifestations, radiographic features, and therapeutic regimens of these patients were retrospectively analysed in the following report.

## Methods

### Study population

The present study was approved by the Tianjin Medical University Eye Hospital Foundation Institutional Review Board (REC No.2017KY(L)L-56) and adhered to HIPAA regulations as well as the principles of the Declaration of Helsinki. The six patients included in this study were selected from 162 cases with a pathological diagnosis of orbital meningioma at Tianjin Medical University Eye Hospital during a 217-month period between August 1999 and September 2017. Patients with known optic nerve sheath meningiomas and intracranial meningiomas were excluded.

### Data collection

Data were collected on patient symptoms, such as headache, nausea, vomiting, and other intracranial symptoms, and the results of regular eye examination, including (i) a visual acuity and best corrected visual acuity test using international visual chart; (ii) examination of the exophthalmos by a Hertel exophthalmometer (differences in the bilateral exophthalmos of more than 2 mm were regarded as abnormal); (iii) examination of eye movement and periorbital changes; and (iv) indirect ophthalmoscopy to check the fundus after mydriasis. All patients underwent radiographic examination, including computed tomography (CT) or magnetic resonance imaging (MRI), to identify the location of their tumour and relative location to the optic nerve, extraocular muscle, and other peripheral tissues.

### Therapeutic regimen and pathological diagnosis

Surgery was the preferred therapeutic regimen. All patients underwent complete surgical resection, and surgical approaches were divided into lateral orbitotomy or anterior orbitotomy according to lesion location. All tumour specimens were sent for pathological examination. The two-step method for immunohistochemical staining was employed to detect the expression of EMA, vimentin, S-100, Ki-67 and calponin and was performed according to the manufacturer’s instructions (Shanghai Bioleaf Biotech Co, Ltd., Shanghai, China). Phosphate buffered saline (PBS) was used as the negative antibody control, and the EMA antibody for clinical pathology diagnosis was used as the positive control. Diaminobenzidine (DAB)-staining, haematoxylin staining, dehydration, transparentisation and sealing with neutral balsam were performed in that order. Positive staining presented as a tan colour in staining assessment.

## Results

### Characteristics of the study population and their medical conditions

All patients were diagnosed with monocular diseases. Among them, four were male, and two were female, with a male to female ratio of 2:1. The mean age at first visit and age range were 33.2 and 7 to 56 years, respectively; the mean disease history and range were 20.3 and 3 to 72 months, respectively. The main complaints recorded at the first visit were upper eyelid oedema (6/6, 100%), exophthalmos (5/6, 83%), ptosis (4/6, 66.7%), impaired vision (2/6, 33%), diplopia (2/6, 33%) and tumours detected by physical examination (2/6, 33%). Three patients developed intracranial symptoms; two of whom had symptoms of nausea and vomiting due to diplopia. One such patient had a headache in accordance with a history of migraines for many years. Two patients had a history of remote head trauma, but this was considered unrelated to their intracranial symptoms. Patients were misdiagnosed as having neurofibromatosis (one case), eosinophilic granuloma (one case), venous haemangioma (one case), and capillary haemangioma (two cases) (Table 1).

**Table 1:** Clinical manifestations in six patients with ectopic orbital meningioma

No.	Age/Sex	History (months)	Visual acuity	Head trauma	Exophthalmos (mm)	Ptosis	Upper eyelid edema	Mobility restriction/ diplopia	Fundus abnormality	Intraorbital pressure	Initial misdiagnosis	Treatment	prognosis
1	7/M	5	1.2	–	14 (95)11	–	+	+	–	–	capillary haemangioma	surgical resection	No recurrence
2	18/F	24	1.2	–	22.5 (105)14.5	+	+	+	–	–	capillary haemangioma	surgical resection	No recurrence
3	31/M	12	LP	+	14 (109)13	–	+	+	Papilledema and increased cup-disc ratio	+	venous haemangioma	surgical resection	No recurrence
4	35/M	72	1.0	+	21 (98)13	+	+	+	papilledema	–	eosinophilic granuloma	surgical resection	No recurrence
5	56/M	3	1.0	–	16.5 (93)13	+	+	+	–	+	undiagnosed	surgical resection	No recurrence
6	52/F	6	0.5	–	18 (105)13	+	+	+	–	+	neurofibromatosis	surgical resection	

### Ophthalmic manifestations

In most cases, the visual acuity of patients was better than 1.0 (4/6, 67%). All cases were observed to have different degrees of unilateral exophthalmos (Additional file 1: Figure. a and c). Other ophthalmic manifestations included upper eyelid oedema, mobility restriction, light diplopia, different levels of increased intraorbital pressure, and fundus abnormalities, including papilledema and optic nerve compression with an increased cup-disc ratio (Table 1).

### Radiographic features

**Fig. 1:**
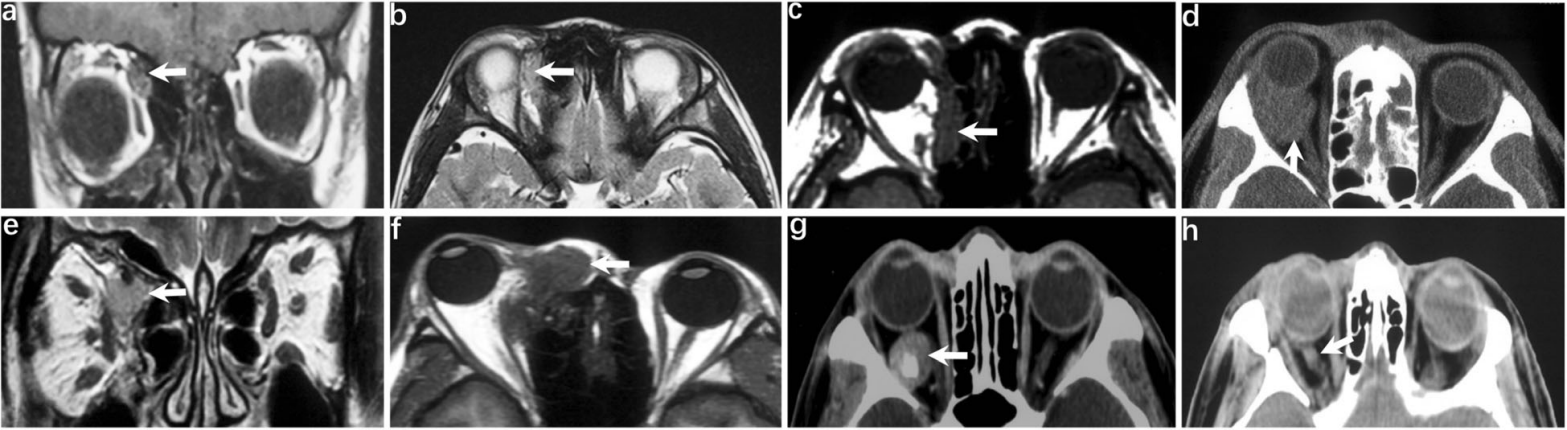
**a** and **b** MRI of case 1. **a** coronal T1WI showing the superonasal mass (arrow). **b** Axial T2WI showing an ill-defined and heterogeneous mass and adjacent medial rectus (arrow). **c** MRI of case 2. Axial T1 showing an ill-defined and heterogeneous superonasal mass and adjacent medial rectus (arrow). **d** Axial CT of case 3. A well-defined intraconal mass adjacent to the anterior optic nerve (arrow). **e** and **f** MRI of case 4. **e** Coronal T1 W1 showing the superonasal mass and no adjacent medial rectus (arrow). **f** Axial T1 W1 showing the ill-defined and heterogeneous superonasal mass (arrow). **g** and **h**: CT of case 5. **g** Axial CT showing a well-defined intraconal lesion with a calcified mass (arrow). **h** Optic nerve was compressed and dislocated but integrated into the structure (arrow)

All cases underwent either CT or MRI examination. CT examination was indicative of ill-defined and heterogeneous lesions, with calcium spots present in one case. Most cases were recognised as having neither optic nerve nor sphenoid ridge involvement. Some cases were observed to have tumours in close proximity to the optic nerve. These were likely to be mistaken as originating from the optic nerve because the human eye is limited when identifying CT values. Indeed, completely preserved optic nerves were observed in all cases after surgery, and the periosteal nerve had no proliferation and no bone involvement. T1WI MRI was hypointense and T2WI MRI was hyperintense in all cases. CT and MRI images are shown in Fig. 1.

### Therapeutic regimen and pathological diagnosis

All cases underwent complete surgical resection. The following surgical methods were used: four cases of lateral orbitotomy, one case of transconjunctival orbitotomy, and one case of supraorbital orbitotomy. During surgery, the optic nerves remained intact while tumour resection was performed, and no orbital bone involvement or periosteal proliferation was observed. However, most tumours were observed as having different degrees of adhesion with extraocular muscles, including the medial rectus (two cases), lateral rectus (two cases), both medial rectus and lateral rectus (two cases), and both medial rectus and superior oblique (one case).

**Fig. 2:**
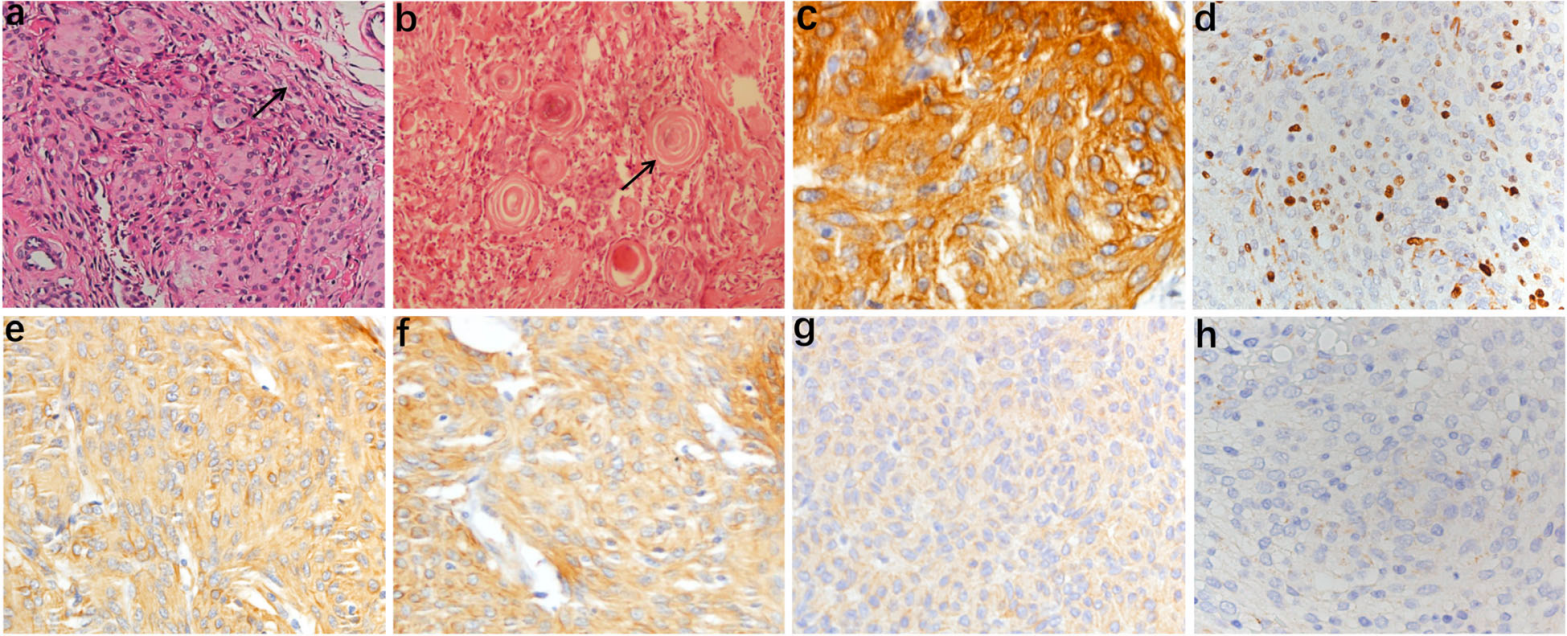
**a** Epithelial-type meningioma with low malignancy. Tumours indicated an infiltrative growth pattern with invasion of surrounding adipose tissue (arrow), HE× 40. **b** Image of psammomatous meningioma, HE× 200. **c-g** immunohistochemistry revealed positive staining for EMA, Ki-67, Vimentin, S-100 and calponin respectively, IHC × 200. **h** PBS was used to replace the primary antibody as the negative control, IHC × 200

The postoperative histopathologic classification of five cases revealed meningothelial cells, and one case revealed psammomatous meningioma (Fig. 2b). In the WHO grading system, the tumours of all six cases were considered to be grade I[5]. However, case NO.1 was considered to have low malignancy because of its invasion of surrounding adipose tissue (Fig. 2a). All cases underwent immunohistochemistry (IHC) (Table 2). IHC revealed EMA and vimentin to be positive (Fig. 2c and e); Ki-67 levels of all cases were less than 3% (Fig. 2d). S-100 was expressed in the two youngest cases, which showed low malignancy (Fig. 2f); Two patients had calponin expression (Fig. 2g). Calponin is an actin-binding protein, and there is clear evidence from previous reports that calponin is strongly expressed by meningeal cells from the lamina propria of the olfactory bulb (OB) [6]. Two patients in this report had calponin expression, and both of these tumours were located in the superonasal extraconal compartment. For this reason, we speculated that meningeal cells were supposed to pass through the frontoethmoidal suture to the orbit and grow into tumours.

**Table 2:** Tumors with immunohischemistry in different locations

NO.	Location	EMA	Vimetin	S-100	Calponin	Ki-67
1	SEC	+	+	+	+	2%
2	SEC	+	+	+	–	0.5%
3	IC	+	+	–	–	0.2%
4	SEC	+	+	–	+	0.7%
5	IC	+	+	–	–	0.8%
6	BEC	+	+	–	–	1%

*SEC* superonasal extraconal compartment, *IC* intraconal compartment, *BEC* bitamporal extraconal compartment

After surgeries, all patients’ exophthalmos was obviously relieved (Additional file 1: Figure. b and d). During the follow-up period (1–72 months), no postoperative diminution of vision was noted, and no recurrence was observed.

## Discussion

The existence of ectopic orbital meningiomas is still debated in the field of ophthalmology. Some previous cases have likely been diagnosed as central nervous system or atypical optic nerve sheath meningiomas, so ectopic orbital meningiomas may be underreported.

There is no definitive evidence as to the origin of ectopic orbital meningiomas. One theory, advanced by Craig and Cogela [7], states that no meningeal tissue in normal orbits other than the arachnoid of the optic nerve should be observed after inspecting several autopsy specimens histologically. It was also suggested that ectopic orbital meningiomas originate from the optic nerve sheath and migrate to ectopic locations. Tan and Lim [8] suggested that ectopic orbital meningiomas could originate from the arachnoid sheath of the cranial nerves, as opposed to the optic nerve, in orbit. Furthermore, there is no evidence that arachnoid courses with the cranial nerves into the orbit, in which cases the involved arachnoid tissue must originate outside the orbit. Another theory suggested that ectopic orbital meningiomas may originate from a regressed orbital meningocele or from meningeal tissue trapped outside the centre [9]. Irwin Tendler et al. [10] reported a case involving the sinus and proposed sinus enlargement as a marker of a congenital event that displaced meningeal cells. This may have caused the formation of an ectopic lesion or mechanical stress induced by the presence of an ectopic orbital tumour, thereby causing sinus asymmetry. However, obvious sinus enlargement was not observed in the present study.

In our study, we hypothesised that some ectopic meningiomas originate from meningeal cells of the OB, in which case meningeal cells could pass through the frontoethmoidal suture to the orbit. EMA and vimentin are important markers of meningioma cells, and these proteins were strongly expressed by tumour cells in all cases in the present study. However, we also found only two cases positive for calponin in tumours which were just in the location of the lateral antorbital frontoethmoidal suture. Interestingly, calponin has been reported to be strongly expressed by connective tissue cells, mesenchymal-derived cells, fibroblasts and meningeal cells from the lamina propria of the olfactory mucosa (OM) and the OB [5, 11, 12].

To date, only 20 cases are described. Among them, 14 cases are from other studies in the literature, and the six cases presented here were treated at our hospital over the last 18 years. Among these cases, the male to female ratio and the mean age at presentation were 11:9 and 32.6 years (range 7–77 years), respectively. This is a marked difference from typical meningiomas, where females are more commonly affected, with detection occurring in the fourth or fifth decade of life. The tumour itself was observed to have little impact on vision, as most visual impairment was caused by excessive tumour growth leading to optic nerve compression (4/20, 20%). This finding differs from nerve sheath meningioma, which affects vision early in its development.

All cases presented here were identified during surgery. Remarkably, the tumours from 11 cases, including three cases in our study and eight cases in previous reports, were located in the superonasal extraconal compartment near the frontoethmoidal suture (11/20, 55%). Two cases reported in the previous literature were noted to have neither CT nor MRI data because they were diagnosed before these radiographic instruments came into use. Among the other 18 cases, radiographic features in most cases were ill-defined, heterogeneous orbital masses (15/18, 83%). MRI showed T1WI as hypointense and T2W as hyperintense fat suppression signal enhancement. Some cases of CT indicated calcium spots (4/18, 22%), and recurrence was rare with complete excision (2/20, 10%). Finally, some of the tumours were obviously separated from the optic nerve, and no evidence suggested bony hyperostosis (Table 3) [3, 4, 8–10, 13–17].

**Table 3:** Results of our 6 cases and 14 cases from literature review of ectopic orbital meningioma

Clinical Characteristics	Literature Review (*N* = 14)	Our Data (*N* = 6)	Total Data (*N* = 20)
Sex			
Male	7	4	11 (55%)
Female	7	2	9 (45%)
Range of age(years)(mean)	7–77 (32.4)	7–56 (33.2)	7–77 (32.6)
History(months)	6–60 (22.4)	3–72 (20.3)	3–72 (20.8)
History of head trauma	2	2	4 (20%)
Symptoms or sign			
Exophthalmos	9	5	14 (70%)
Ptosis	2	4	6 (30%)
Upper eyelid edema	3	6	9 (45%)
Mobility restriction	4	6	10 (50%)
Fundus abnormality	2	2	4 (20%)
Tumor locations			
Superonasal extraconal compartment	8	3	11 (55%)
Bitamporal extraconal compartment	1	1	2 (10%)
Intraconal compartment	5	2	7 (35%)
CT and MRI			
Ill-defined	9	4	13 (65%)
Well-defined	3	2	5 (25%)
Calcification	3	1	4 (20%)
Therapeutic regimen			
Complete resection	12	6	18 (90%)
Radiotherapy	2	0	2 (10%)
Histopathology			
Meningothelial meningioma	12	5	17 (85%)
Fibrous meningioma	2	0	2 (10%)
Psammomatous meningioma	0	1	1 (5%)

Although the cases outlined here did not have a definite diagnosis before pathological testing, our study may offer ophthalmologists cues to improve the diagnostic accuracy for future patients. We found that most patients with ectopic orbital meningioma had upper eyelid oedema and eye mobility restriction through this 18-year clinical retrospective analysis, even though most of the tumours did not involve the eyelids or cause increased intraorbital pressure resulting in obstruction of the returning fluid to the lower eyelid. Such findings are not particularly common in other orbital tumours and may be related to some unknown properties of meningioma cells.

## Conclusions

In summary, orbital isolate lesions, especially around the location of the frontoethmoidal suture, had accompanying upper eyelid oedema and eye mobility restriction not observed in other orbital tumours. Therefore, ectopic orbital meningioma should be considered in such cases. Ideally, further research into the origin and pathogenesis of ectopic orbital meningiomas should be conducted.

## Additional file

Additional file 1: Preoperative and postoperative appearances of two patients. (PDF 5791 kb)

## Abbreviations

CT: Computed tomography
MRI: Magnetic resonance imaging
OB: Olfactory bulb
OM: Olfactory mucosa

## References

[1] Eggers H, Jakobiec FA, Jones IS (1976). Tumors of the optic nerve. Doc Ophthalmol.

[2] Fortuna A, Nicole S, Palma L, Di Lorenzo N (1978). Primary intraorbital meningiomas. Riv Neurol.

[3] Johnson TE, Weatherhead RG, Nasr AM, Siqueira EB (1993). Ectopic (extradural) meningioma of the orbit: a report of two cases in children. J Pediatr Ophthalmol Strabismus.

[4] Pushker N, Shrey D, Kashyap S, Sen S, Khurana S, Sharma S (2013). Ectopic meningioma of the orbit. Int Ophthalmol.

[5] Louis DN, Ohgaki H, Wiestler OD (2007). World Health Organization classification of tumours of the central nervous system.

[6] Ibanez C, Ito D, Zawadzka M, Jeffery ND, Franklin RJ (2007). Calponin is expressed by fibroblasts and meningeal cells but not olfactory ensheathing cells in the adult peripheral olfactory system. Glia.

[7] Craig Winchell Mck., Gogela Louis J. (1949). Intraorbital Meningiomas a Clinicopathologic Study*. American Journal of Ophthalmology.

[8] Tan KK, Lim AS (1965). Primary extradural intra-orbital meningioma in a Chinese girl. Br J Ophthalmol.

[9] Farah SE, Konrad H, Huang DT, Geist CE (1999). Ectopic orbital meningioma: a case report and review. Ophthal Plast Reconstr Surg.

[10] Tendler I, Belinsky I, Abramson DH, Marr BP (2017). Primary Extradural Ectopic Orbital Meningioma. Ophthal Plast Reconstr Surg.

[11] Tome M, Siladzic E, Santos-Silva A, Barnett SC (2007). Calponin is expressed by subpopulations of connective tissue cells but not olfactory ensheathing cells in the neonatal olfactory mucosa. BMC Neurosci.

[12] Rizek PN, Kawaja MD (2006). Cultures of rat olfactory ensheathing cells are contaminated with Schwann cells. Neuroreport.

[13] Yokoyama T, Nishizawa S, Sugiyama K (1999). Primary intraorbital ectopic meningioma. Skull Base Surg.

[14] Arai H, Sato K, Matsumoto T (1997). Free-lying ectopic meningioma within the orbit. Br J Neurosurg.

[15] Decock CE, Kataria S, Breusegem CM, Van Den Broecke CM, Claerhout IJ (2009). Ectopic meningioma anterior to the lacrimal gland fossa. Ophthal Plast Reconstr Surg.

[16] Gunduz K, Kurt RA, Erden E (2014). Ectopic orbital meningioma: report of two cases and literature review. Surv Ophthalmol.

[17] Wolter JR, Benz SC (1976). Ectopic meningioma of the superior orbital rim. Arch Ophthalmol.

