# Supplementary figures and images for: Ectopic orbital meningioma: a retrospective case series
Source: BMC Ophthalmol. 2018 Nov 12;18:296. doi: 10.1186/s12886-018-0959-z (PMC6233548; doi:10.1186/s12886-018-0959-z)

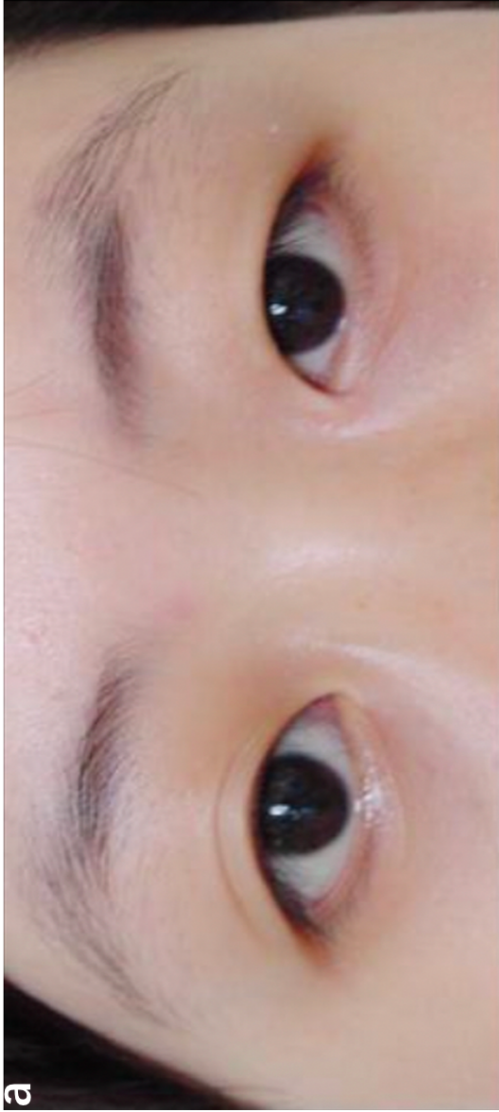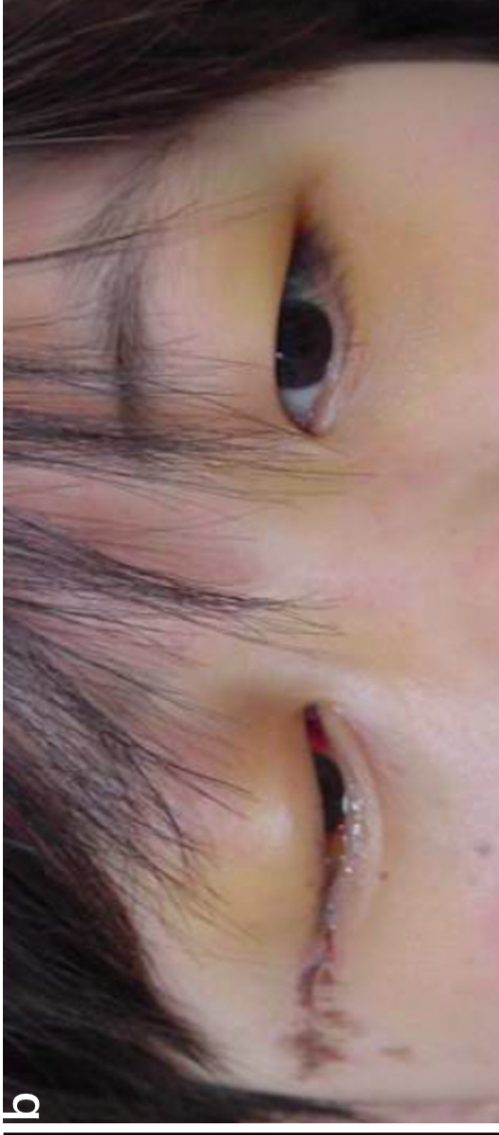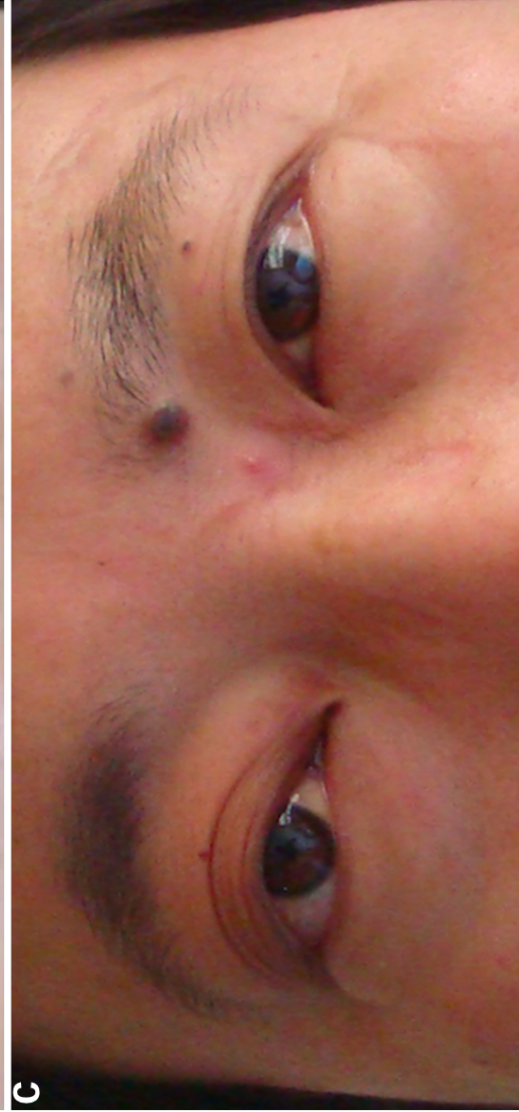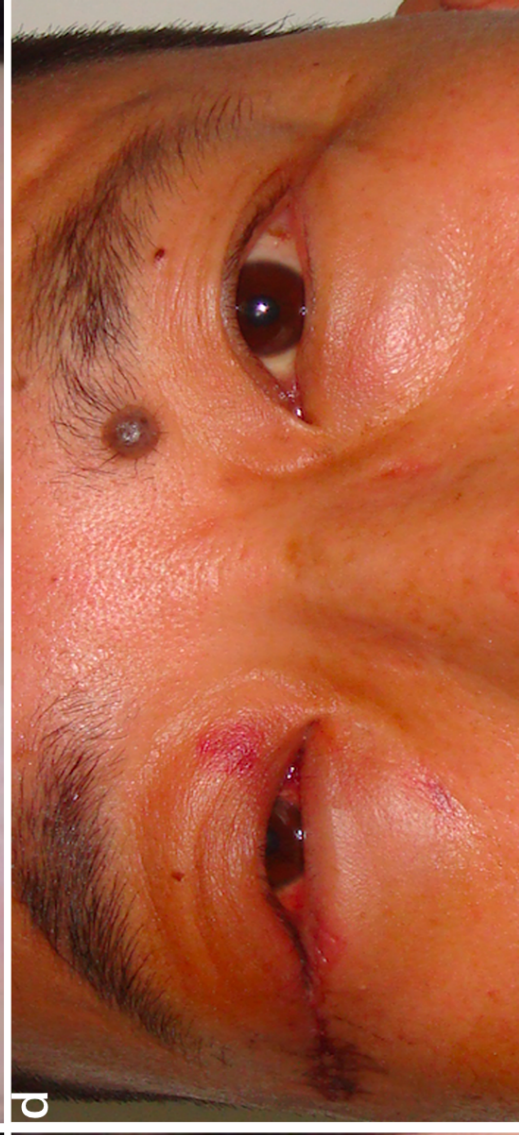

Supplement: Supplementary file 1 — Preoperative and postoperative appearances of two patients. (PDF 5791 kb) [file 12886_2018_959_MOESM1_ESM.pdf]
